# Comparison of the therapeutic effects of surgery following prism adaptation test versus surgery alone in acute acquired comitant esotropia

**DOI:** 10.1186/s12886-020-01574-y

**Published:** 2020-07-23

**Authors:** Peng Zhang, Ying Zhang, Lei Gao, Jun Yang

**Affiliations:** Department of Ophthalmology, Jinan 2nd People’s Hospital, No.148, Jingyi Road, Huaiyin District, Jinan, 250001 China

**Keywords:** Acute acquired comitant esotropia, Deviation angles, Prism adaptation test, Visual function

## Abstract

**Background:**

To compare the therapeutic effects of surgery following prism adaptation test versus surgery alone in acute acquired comitant esotropia (AACE).

**Methods:**

A total of 46 patients with AACE were enrolled in this retrospective study. Among them, 26 patients underwent surgery following prism adaptation test (combination group) and 20 patients underwent surgery alone (surgery group). The following parameters were evaluated including success rate, distant and near deviation angles, visual function, and near stereoacuity.

**Results:**

There were no significant differences in success rate between the combination group and surgery group at post-treatment 12 months (96.15% vs. 90.00%, *p* > 0.05). The postoperative distant and near deviation angles in two groups were significantly lower than that before surgery (*p* < 0.05). In addition, the numbers of patients with stereopsis postoperatively in two groups were significantly higher than that before surgery (all *p* < 0.05). Moreover, the numbers of patients with stereopsis and central stereopsis in the combination group were significantly higher than that in the surgery group postoperatively. At post-treatment 12 months, one (3.85%) case recurred in combination group and three (15.00%) cases in the surgery group. No complications were observed in the two groups.

**Conclusions:**

Both approaches had therapeutic benefit in AACE. Surgery following prism adaptation test had better treatment benefits than surgery alone in improving binocular function and reducing recurrence rate.

## Background

Acute acquired comitant esotropia (AACE) is a rare type of esotropia that usually occurs in older children and adults [1]. It is classified into three types: 1) Swan type: esotropia due to monocular occlusion or loss of vision in one eye; 2) Burian-Franceschetti type: esotropia characterized by minimal hypermetropia and diplopia, often associated with physical or psychological stress; 3) Bielschowsky type: esotropia in patients with varying degrees of myopia, and shows equal deviation at near and distance fixation [2, 3]. Its clinical characteristics include an acute onset of comitant esotropia with diplopia (same deviation in all gaze direction), normal ocular motility, recessive, constant or intermittent esotropia, and a certain binocular visual function [4, 5]. The current treatment strategies are surgery, botulinum toxin injection and prescription of prism glasses [3, 6, 7]. Botulinum toxin has been used to diagnose and treat different types of strabismus since the 1970s [8]. Compared with standard strabismus surgery, botulinum toxin injection has many advantages, such as simple operation, short anesthesia time, short post-anesthesia care time and low cost [9]. However, there is no recommendation for the standardized dose of botulinum toxin based on deviation angle currently. In addition, the recurrence rate of the strabismus after botulinum toxin injection is high, requiring reinjection [10]. These disadvantages limit the widespread use of botulinum toxin in the treatment of strabismus.

The prism adaptation test, which was popularized by Jampolsky in 1971, has become a possible solution for correcting acquired esotropia more accurately and successfully by revealing the maximum angle of deviation that may otherwise be masked [11, 12]. Previous studies have showed that prism correction could improve the success rate of strabismus surgery for patients with esotropia [11, 13]. Therefore, surgical treatment or preoperative prism correction is often used in treatment of AACE currently. However, it is unknown whether there is a difference between the two therapies and which treatment is better for treatment of AACE. In addition, there are few reports about the changes of binocular vision function in patients with AACE after preoperative prism correction. In this study, we aimed to compare the therapeutic effects of surgery following prism adaptation test versus surgery alone in AACE, and to provide a basis for the treatment of AACE.

## Methods

### Patients

Between January 2011 and September 2018, a total of 46 patients with AACE treated with surgery following prism adaptation test (combination group) vs. surgery alone (surgery group) at our hospital were included in this retrospective study. Ethics approval was obtained from the Institutional Review Board of Jinan Second People’s Hospital and informed consent was obtained from each participant and their guardians.

The inclusion criteria were: (1) patients with a diagnosis of AACE; (2) patients with sudden-onset esotropia with diplopia (same deviation in all gaze direction); (3) patients with the corrected visual acuity not less than 20/20 in both eyes; (4) patients with duration of more than 2 months from onset to treatment.

The exclusion criteria were: (1) patients with other eye diseases, such as strabismus and amblyopia; (2) history of eye trauma, medication and surgery; (3) presence of systemic diseases (such as diabetes, myasthenia gravis, etc); (4) history of brain tumor or neurological disease.

### Ophthalmological examination

All patients underwent routine ophthalmologic examinations (refraction, visual acuity, outer eye, anterior segment, fundus), head computed tomography (CT) or magnetic resonance imaging (MRI). As descripted previously, AACE was classified into Swan type, Burian-Franceschetti type and Bielschowsky type [2, 3]. The accommodative convergence/accommodation (AC/A) ratio was measured with the gradient method preoperatively to check for accommodative esotropia. Cycloplegic refraction was performed after administering 1% atropine ointment in patients< 9 years and 1% compound tropicamide eye drops for those≥9 years old. The three grades of binocular visual function including simultaneous perception, fusion and stereopsis were measured by synoptophore (TSJ-IV-A, Photoelectric Instrument Co., Ltd. Changchun, China). The distant (6 m) and near (33 cm) deviation angles was measured by prism and alternating cover test. Near stereoacuity was measured using the Titmus test (Stereo Optical, Chicago, IL) at the standard viewing distance of 40 cm with full correction [14]. Patients were asked to catch the wings of the fly, and then proceed to the row of animals, followed by graded circle test. Patients were asked to point to animal or circle or push the circle which seemed to float up. Following the correct response, patients were shown the next level of targets and the near stereoacuity values were recorded. Therefore, the criterion for recording was successive correct responses at a given disparity level (ranges from 800“ to 40”).

#### Therapeutic methods

During calculation of prism strength, the prisms were equally divided over the left and right eyes in a trial-frame to correct for the esotropia. The combination group was then given prism correction using Fresnel press-on prisms mounted base out over the entire lens on the patient’s spectacle correction within 1 week to 3 months after the onset. The initial prism power was based on amount of near esodeviation while fixating on an adaptive target.

All patients were observed until 6 months after the onset, and surgery could only be performed after the degree of strabismus had stabilized. When the angle of deviation was larger at distance than at near (≥10 PD), it was considered to be divergence insufficiency type of esotropia. In contrast, when the angle of deviation was larger at near than at distance (≥10 PD), it was considered to be convergence excess type of esotropia. Basic type of esotropia was defined as a < 10-PD difference between near and distance deviations. In patients with basic type and convergence excess type of esotropia, our operation of choice was unilateral or bilateral medial rectus recession. In patients with divergence insufficiency type of esotropia, we preferred medial rectus recession and lateral rectus resection. The surgery was performed under general anesthesia in patients< 14 years and local anesthesia for those≥14 years old. The prisms were discontinued after surgery.

### Observational items

The mean preoperative prism correction time was 6.5 ± 1.8 months (range, 3–20 months). Three grades of binocular visual function, near stereoacuity, distant and near deviation angles and complications were measured during the follow-up period (12 months). Success was defined as postoperative total horizontal deviation of 10 PD or less. Near stereoacuity ≤60“ was the central stereopsis, 80”-800“ was peripheral stereopsis, and ≥800” was stereo blindness.

### Statistical analysis

Statistical analysis was performed by IBM SPSS Statistics Version 20.0 (IBM Corp., Armonk, NY, USA). Numerical data were expressed as means±standard deviations (SD) and were compared using Student’s t-test. Categorica data were expressed as number and percentage and were compared using χ^2^ test. Statistical significance was set at *p* < 0.05.

## Results

### Baseline characteristics

A total of 46 patients diagnosed with AACE were retrospectively analysed in this study. Among them, 26 patients including 14 (53.85%) males and 12 (46.15%) females with the mean age of 19.00 ± 11.16 years (range, 4–50 years) underwent surgery following prism adaptation test (combination group), and another 20 patients including 11 (55.00%) males and 9 (45.00%) females with the mean age of 18.80 ± 9.79 years (range, 3–45 years) underwent surgery only (surgery group). The baseline characteristics of included subjects are listed in Table 1. The two groups were generally well balanced for age, sex, time from onset to treatment, refraction and type of AACE (all *p* > 0.05, Table 1).

**Table 1:** Baselines characteristics of included patients

Characteristics	Combination group (*n* = 26)	Surgery group (*n* = 20)	*p* value
Age (years), Mean ± SD/range	19.00 ± 11.16 (4–50)	18.80 ± 9.79 (3–45)	0.950
Gender (no, %), M/F	14 (53.85)/12 (46.15)	11 (55.00)/9 (45.00)	0.983
Time from onset to treatment (mo), Mean ± SD	9.00 ± 3.14	8.95 ± 3.66	0.962
Refraction (D), Mean ± SD			
Right	2.02 ± 3.29	2.29 ± 3.09	0.787
Left	1.86 ± 3.14	2.08 ± 3.05	0.813
Type of AACE			
Burian-Franceschetti	11	10	0.604
Bielschowsky	12	13	0.203

*M* male; *F* female; *no* number; *SD* standard deviations; *D* diopter; *mo* months

In the combination group, 14 patients underwent two muscle surgery and 12 patients underwent single muscle surgery. In the surgery group, 12 patients underwent two muscle surgery and 8 patients underwent single muscle surgery.

### Clinical evaluation outcomes

At post-treatment 12 months, the success rate was 96.15% (25/26) in the combination group and 90.00% (18/20) in the surgery group, and no significant difference between two groups (*p* > 0.05).

The distant and near deviation angles 12 months postoperatively in two groups were significantly lower than that before surgery (all *p* < 0.05), suggesting the effectiveness of these two treatment methods (Table 2). However, there were no significant differences in the distant and near deviation angles between combination group and surgery group postoperatively.

**Table 2:** The deviation angles and visual function in two groups preoperatively and postoperatively

Parameter	Combination group (n = 26)		Surgery group (n = 20)	
	Preoperative	Postoperative	Preoperative	Postoperative
Deviation angles, Mean ± SD				
Distant	39.62 ± 12.48	2.92 ± 2.15^a^	40.05 ± 8.72	4.00 ± 2.51^a^
Near	37.88 ± 11.06	−0.27 ± 2.52^a^	39.00 ± 9.54	0.50 ± 2.26^a^
Visual function, no (%)				
Simultaneous perception	16 (61.54)	24 (92.31)^a^	12 (60.00)	18 (90.00)^a^
Fusion	10 (38.46)	21 (80.77)^a^	6 (30.00)	12 (60.00)
Stereopsis	5 (19.23)	20 (76.92)^a^	3 (15.00)	9 (45.00)^ab^
Near stereoacuity, no (%)				
Central stereopsis	5 (19.23)	22 (84.62)^a^	3 (15.00)	11 (55.00)^ab^
Peripheral stereopsis	13 (50.00)	24 (92.31)^a^	8 (40.00)	17 (85.00)^a^
Stereo blindness	13 (50.00)	2 (7.69)^a^	12 (60.00)	3 (15.00)^a^

^a^ vs. preoperative, *p* < 0.05; ^b^ vs. combination group, *p* < 0.05; no, number; SD, standard deviations

The three grades of binocular visual function (Table 2) results showed that the numbers of patients with simultaneous perception and stereopsis 12 months postoperatively in two groups were significantly higher than that before operation (all *p* < 0.05). In addition, changes in fusion were also observed after post-treatment 12 months, but only the combination group reached statistical significance (*p* < 0.05). Moreover, the numbers of patients with stereopsis in the combination group were significantly higher than that in the surgery group postoperatively (*p* < 0.05). These results indicated surgery following prism adaptation test had better treatment benefits than surgery alone in improving visual function.

As shown in Table 2, the numbers of patients with stereo blindness were all significantly reduced 12 months postoperatively in two groups (*p* < 0.05), but no statistical difference was observed between the two groups. In addition, the numbers of patients with central stereopsis and peripheral stereopsis 12 months postoperatively in two groups were significantly higher than that before operation (all *p* < 0.05). More importantly, the numbers of patients with central stereopsis in the combination group were significantly higher than that in the surgery group postoperatively (*p* < 0.05, Table 2). This suggested that surgery following prism adaptation test had better treatment benefits than surgery alone in improving near stereoacuity.

### Recurrence and complications

At post-treatment 12 months, one (3.85%) case recurred in the combination group and three (15.00%) cases in the surgery group (*p* > 0.05). No complications such as scleral perforation, anterior segment ischemic syndrome, muscle slippage, muscle hematoma, subconjunctival cysts or granulation hyperplasia occurred in either group.

## Discussion

AACE is an unusual presentation of esotropia that occurs in adults and older children [15]. At present, AACE is mostly treated by preoperative prism correction, surgery or botulinum toxin injection [16]. Wan et al. found that botulinum toxin was at least as effective as standard incisional strabismus surgery in the treatment of AACE at 6 months [5]. Velez et al. evaluated the efficacy of preoperative prism adaptation for a longer follow-up period in subjects with acquired esotropia, and found that patients who underwent prism adaptation maintained better motor alignment than those who not underwent prism adaptation over a long-term follow-up period (> 12 months) [17]. However, there was no existing study directly comparing the therapeutic effects of surgery following prism adaptation test versus surgery alone in AACE. In this study, we aimed to compare the therapeutic effects of surgery following prism adaptation test versus surgery alone in AACE.

The preoperative prism adaptation test has been used to determine the target angle for surgical correction and decrease the risk of undercorrection or overcorrection in patients with acquired esotropia undergoing surgical correction [18]. Previous studies have shown that preoperative prism adaptation could improve the success rate of strabismus surgery for patients with esotropia [11, 13]. In the present study, we found that the success rate was 96.15% in the combination group and 90.00% in the surgery group. This is consistent with previous studies. Repka et al. found that success rate of patients with acquired esotropia was 90.0% after preoperative prism adaptation [19, 20]. In addition, Lang et al. reported that the success rate of patients with AACE was 81.3% postoperatively [6]. Moreover, we found that postoperative distant and near deviation angles in two groups were significantly lower than that before operation. However, there were no significant differences in the distant and near deviation angles between two groups postoperatively. These results indicated that surgery following prism adaptation test was comparable to surgery alone in success rate and deviation angle.

Campos et al. reported that patients with Bielschonsky type AACE had a tendency to continuous binocular convergence and relapse after surgery [21]. In this study, we found that one case recurred 12 months postoperatively in the combination group and three cases in the surgery group. Consistent with previous study, all patients who relapsed were Bielschonsky type AACE. In addition, we found that the recurrence rate in the combination group was obviously lower than that in the surgery group (3.85% vs. 15.00%), but no significant difference between the two groups (*p* > 0.05). The possible reason was that the number of samples in this experiment was small and the follow-up time was short and requires further research.

In the present study, we also found that 84.62% cases developed near stereoacuity ≤60“ in the combination group, and 55.00% cases developed near stereoacuity ≤60” in the surgery group postoperatively. Spierer et al. found that stereoacuity was 40″ in all adult patients after surgery [22], which was different from our reports. It may be related to the binocular visual function of adult patients had developed normally before the onset of esotropia [23]. Therefore, it could recover after strabismus surgery. In the present study, we also found that the three grades of binocular visual function (simultaneous perception, fusion and stereopsis) postoperatively in two groups were higher than that before operation. Otherwise, the stereopsis and near stereoacuity in the combination group were better than that in the surgery group postoperatively, which indicated that preoperative prism correction for patients with AACE was very important for the maintenance and recovery of binocular visual function.

There were several limitations in the present study. Firstly, this study was a retrospective study conducted at a single institute which had certain limitations in clinical application. Secondly, the number of patients included in this study was small. Thirdly, it was necessary to have a longer follow-up in the surgery following prism adaptation test group to evaluate further deterioration, especially for patients with high degree of strabismus. Fourthly, the decision of whether to proceed with prism correction plus surgery or single surgery as the primary treatment was based on patient selection and physician preference.

## Conclusions

Both approaches had therapeutic benefit in AACE. Surgery following prism adaptation test had better treatment benefits than surgery alone in improving binocular function and reducing recurrence rate.

## Data Availability

Data is obtained with the permission of the corresponding author.

## Abbreviations

AACE: Acute acquired comitant esotropia
CT: Computed tomography
MRI: Magnetic resonance imaging
SD: Standard deviations
